# Model-Selection Theory: The Need for a More Nuanced Picture of Use-Novelty and Double-Counting

**DOI:** 10.1093/bjps/axw024

**Published:** 2016-08-30

**Authors:** Katie Steele, Charlotte Werndl

**Affiliations:** 1 Department of Philosophy, Logic, and Scientific Method London School of Economics London, UK and School of Philosophy Research School of the Social Sciences (RSSS) College of Arts and Social Sciences Australian National University Canberra, Australia; 2 Department of Philosophy (KGW) University of Salzburg Salzburg, Austria Department of Philosophy, Logic, and Scientific Method London School of Economics London, UK

## Abstract

This article argues that common intuitions regarding (a) the specialness of ‘use-novel’ data for confirmation and (b) that this specialness implies the ‘no-double-counting rule’, which says that data used in ‘constructing’ (calibrating) a model cannot also play a role in confirming the model’s predictions, are too crude. The intuitions in question are pertinent in all the sciences, but we appeal to a climate science case study to illustrate what is at stake. Our strategy is to analyse the intuitive claims in light of prominent accounts of confirmation of model predictions. We show that on the Bayesian account of confirmation, and also on the standard classical hypothesis-testing account, claims (a) and (b) are not generally true; but for some select cases, it is possible to distinguish data used for calibration from use-novel data, where only the latter confirm. The more specialized classical model-selection methods, on the other hand, uphold a nuanced version of claim (a), but this comes apart from (b), which must be rejected in favour of a more refined account of the relationship between calibration and confirmation. Thus, depending on the framework of confirmation, either the scope or the simplicity of the intuitive position must be revised.

**1** *Introduction*
**2** *A Climate Case Study*
**3** *The Bayesian Method vis-à-vis Intuitions*
**4** *Classical Tests vis-à-vis Intuitions*
**5** *Classical Model-Selection Methods vis-à-vis Intuitions*
    **5.1** *Introducing classical model-selection methods*
    **5.2** *Two cases*
**6** *Re-examining Our Case Study*
**7** *Conclusion*

## 1 Introduction

Scientists and philosophers alike have the intuition that the empirical fit of a model or theory—that is, how well the theory accords with relevant data—has lesser significance for the predictive reliability or truth of the theory, to the extent that the theory was designed to accommodate the data in question. For example, empirical fit with observed retrogressions of planets was considered less indicative of the reliability of Ptolemy’s theory as compared to Copernicus’s theory given that the former, as opposed to the latter, was refined specifically to account for the observational data about retrogressions (cf. Lakatos and Zahar [1976]). The puzzle arises, however, as to whether these intuitions are vindicated by a plausible account of confirmation and, if so, how any such account explains the differential power of predicted (and thus use-novel), as opposed to accommodated, data in giving support to or, in other words, confirming the reliability of a theory.

John Worrall ([2010]) goes a long way towards meeting this challenge.^1^ Rather than appealing to broad psychological tendencies amongst scientists or to properties of data, such as time of discovery, that surely do not have a direct bearing on confirmation, Worrall offers an account of use-novelty that rests on the relationship, in terms of actual empirical content, between theory and data (cf. Musgrave’s ([1974]) distinction between logical and historical theories of confirmation). Worrall casts use-novelty as turning on whether data are used to settle on a specific version of a general theory or, in other words, whether data are used to settle the free parameters of a general (or base) theory. Data that are not used to settle free parameters are predicted data as opposed to accommodated data, and thus when it comes to confirmation, such data are use-novel.

The significance of use-novelty for Worrall is summed up in his ‘no-double-counting’ rule, which effectively says that data used in settling the free parameters of a general theory cannot play a role in adding support or incrementally confirming the theory’s truth or reliability. More precisely, while data used to fix free parameters provide support for the resulting version (or instance) of a general theory relative to other versions that fit the data less well, this support does not ‘spread’ to the general theory itself. In other words, data used to fix free parameters provide support for the calibrated theory conditional on the general theory being correct, but these data do not provide unconditional support for the calibrated (or the general) theory. This account provides an effective way of conceiving the difference between, for instance, the Copernican and Ptolemaic theories, *vis-à-vis* the observational data about retrogressions. In the case of the Ptolemaic theory, the data are used to settle on a specific instance of the theory that can fit the data about retrogressions; while it follows from the basic geometry of the Copernican theory that there are retrogressions, and hence there is a qualitative fit to the data about retrogressions without further refinements of the Copernican theory (Lakatos and Zahar [1976]). Intuitively, there is no confirmation of the general Ptolemaic theory in this case (albeit confirmation of a specific version of Ptolemaic theory relative to others), while there is indeed confirmation of the general Copernican theory. Furthermore, given the emphasis on incremental rather than absolute confirmation, Worrall’s account succeeds in explaining the differing standing of theories constructed to accommodate data. While neither the Ptolemaic theory nor, say, various Intelligent Design theories are incrementally confirmed by data used to construct or calibrate them, the former is simply more credible from the outset, since the basic form of the Ptolemaic theory, unlike Intelligent Design, is supported by other data and also by theoretical considerations.

This article examines the cogency of Worrall’s account when it is applied broadly. Calibration is a widespread phenomenon in science, and the question is whether Worrall’s overarching maxims concerning calibration and confirmation are themselves plausible across a diversity of cases. While appealing to the intuitions of scientists and philosophers alike, does Worrall’s account of use-novelty and his associated no-double-counting rule (henceforth, ‘the intuitive position’) make sense in the full range of confirmation contexts?

We appeal to a case study from climate science, illustrative of the more standard model-building problem in science, as the platform from which to examine Worrall’s claims. This case study is outlined in Section 2. Subsequent sections assess the intuitive position in light of prominent accounts of evidence and confirmation. Our primary goal is to challenge intuitions about use-novelty and its significance for confirmation by exposing just how varied the stances of prominent theories of confirmation are on this issue. Others have critiqued Worrall’s claims about use-novelty from the perspective of one or another theory of confirmation: notably, Howson ([1988]), Steele and Werndl ([2013]), and Schurz ([2014]) give more or less charitable Bayesian critiques of Worrall’s claims, Mayo (for instance [1991], [2014]) compares Worrall’s views with the tenets of ‘severe testing’, and Hitchcock and Sober ([2004]) evaluate Worrall’s claims by appeal to the standards of model-selection theory. We cite this work where relevant in our discussion. The importance of our contribution here is the comparative treatment of different theories of confirmation. We show that intuitions about use-novelty such as Worrall’s do not clearly favour any particular theory of confirmation. This is not intended as an argument for pluralism about confirmation; it is, more modestly, an argument for caution in assessing the normative significance of intuitions about confirmation.

Following the presentation of our case study, the article proceeds as follows: Section 3 considers the Bayesian perspective, and shows why it does not generally uphold the intuitive position, despite permitting a distinction between data for calibration and use-novel, confirming data in certain types of cases. Section 4 presents the simplest classical approach to assessing models, the hypothesis-testing method, which is shown to be very similar to the Bayesian method when it comes to use-novelty and double-counting. Section 5 turns to the more specialized classical model-selection methods. These methods affirm the specialness of use-novelty—or, at least, they explicitly account for the related danger of over-fitting models to data, and so partially conform to the intuitive position, but they do not uphold the link between use-novelty and no-double-counting. We return to the climate case study in Section 6, drawing attention to the dilemma posed in the article for extending and reforming the intuitive position.

## 2 A Climate Case Study

Willett *et al.* ([2007]) are concerned with explaining the increase of surface-specific humidity (the mass of water vapour in a unit mass of moist air) in the past decades. More specifically, they compare the performance of two base models as an explanation of this trend. The first base model, *M*_1_, is the third Hadley Centre coupled model with a linear combination of anthropocentric forcings and natural forcings. The second base model, *M*_2_, is the third Hadley Centre coupled model where only natural forcings are considered.

There is a specific spatio-temporal pattern of change (a fingerprint) associated with the anthropocentric forcings and a specific spatio-temporal pattern of change associated with the natural forcings. While this fingerprint is given, what is not known is the extent of the response to the forcings. Thus the extent of the response corresponds to free parameters in the models that have to be estimated from the data. That is, in the case of base model *M*_1_, there are two free parameters (one that measures the extent of the response to the anthropocentric forcings and one that measures the extent of the response to the natural forcings), and model instances of *M*_1_ are obtained when the two parameters are assigned specific values. For model *M*_2_, there is only one free parameter (that measures the extent of the response to the natural forcings) and, again, model instances of *M*_2_ are obtained when the free parameter is assigned a specific value.

Willett *et al.*’s data consist of observations of surface-relative humidity changes from 1973 to 2003. The squared distance between simulations and observations is used to measure the fit of the observations with the model instances. Willett *et al.* first use the data about surface humidity to estimate the parameter values of *M*_1_. The best estimates for the extent of the anthropocentric forcing and natural forcing, respectively, are (1.12, 2.22). The 95% confidence intervals that Willett *et al.* also report reveal that both parameter values are significantly different from zero. From this, Willett *et al.* conclude that none of the model instances of *M*_2_ provides a satisfactory fit with the data about relative humidity, and thus that base model *M*_1_ is confirmed relative to base model *M*_2_. They also emphasize that this is the first demonstration that specific humidity trends can only be explained with both natural and anthropocentric forcings.

To sum up, Willett *et al.* use data about surface-relative humidity to estimate the values of the free parameters (calibration). At the same time, they use the same data to confirm base model *M*_1_ relative to base model *M*_2_. Philosophers as well as climate scientists have often debated whether such a procedure is legitimate. More specifically, one question is whether data have to be use-novel, that is, whether data can only be used for confirmation if they have not already been used before for calibrating the free parameters. A second and related question is whether it is permissible to use the same data both for calibration and confirmation (in this case, one says that there is double-counting). Note that it is just an accidental feature of the base models in the case study that they are nested base models. The questions of use-novelty and double-counting arise whenever two base models are compared, whether they are nested base models or not. Indeed, in the next section we will compare two base models, *M* and *N*, that are not nested.

## 3 The Bayesian Method *vis-à-vis* Intuitions

This section focuses on what Bayesian logic of confirmation and evidence has to say about the questions of use-novelty and double-counting posed above. For the Bayesian, what is at issue for model confirmation is the truth of a proposition, in this case a hypothesis describing the model prediction(s). The extent of confirmation of the truth of the hypothesis is measured in terms of probability. Typically, the Bayesian is interested in the change in the probability of the truth of a hypothesis due to new data, that is, incremental confirmation.

For ease of exposition and because it permits a clearer analysis, we will shortly introduce illustrative models that are simpler than those employed in the climate science case study. We will see that, while the Bayesian framework permits a formal depiction of calibration very much in line with Worrall’s account, it does not vindicate the intuitive position in its general form (cf. Steele and Werndl [2013]). Having said that, something of the position can be salvaged for a restricted set of cases.

Consider the following two base models:

$$\begin{array}{l} M:z(x,y)=a\cdot x+b\cdot y+c+N(0,\sigma ),\\ N:z(x,y)=d\cdot x+e\cdot y^{2}+f+N(0,\sigma ). \end{array}$$

Here *σ* is given; it is the standard deviation for the Gaussian error term. On the other hand, *a*, *b*, *c*, *d*, *e*, and *f* are uncertain, with each assumed to be in the range −10–10. (For simplicity of exposition, we restrict our attention to the discrete case where the uncertain parameters may take only values from a finite set within this range.)

In line with Worrall’s account of calibration, the Bayesian treats a base model as a set of fully specified model hypotheses, one hypothesis for each possible instantiation of the base model or, in other words, one hypothesis for each possible combination of free-parameter values for the base model. For our example, assume that each of these model-instance hypotheses claims to give the value of *z*, as would be observed in the real world or, rather, a probability distribution for the observed *z*. Note that the probabilistic error term may account for observational error, that is, the discrepancy between the true value of *z*, as predicted by the model, and the observed *z*. It may otherwise account for model structural error in predicting the true value of *z*.

For the case we are considering, there are two base models, *M* and *N*, corresponding to two sets of model-instance hypotheses, which may be labelled *M_a_*_,__*b*__,__*c*_ and *N_d_*_,__*e*__,__*f*_, where the subscripts indicate the values of the parameters for the model instance in question. The scientist’s initial assessment of the truth of the various hypotheses is represented by a prior probability distribution over these hypotheses. (In fact, the prior probability distribution extends to all propositions in the domain, a Boolean algebra, including all hypotheses and potential evidence.)

For the Bayesian, calibration is not really distinct from confirmation. When new data are learnt, the relevant evidence proposition is effectively assigned a probability of one, and the probabilities for all other propositions in the domain are updated according to the Bayesian rule of conditionalization. This rule can be stated in terms of a ratio of the new probabilities, *Pr*_new,_ of any proposition, *H*, relative to some other proposition, H′, where *E* is the evidence or the data learnt, and *Pr* is the initial or prior probability distribution:

(1) $$\frac{Pr_{\text{new}}(H)}{Pr_{\text{new}}(H\prime )}=\frac{Pr(H|E)}{Pr(H\prime |E)}=\frac{Pr(E|H)}{Pr(E|H\prime )}\cdot \frac{Pr(H)}{Pr(H\prime )}.$$

The term $\frac{Pr(E|H)}{Pr(E|H\prime )}$ is referred to as the likelihood ratio. If this term in greater than one, then *H* is more incrementally confirmed than H′ by evidence *E*. If it is less than one, then *H* is disconfirmed relative to H′, and if it is equal to one, then *H* is neither confirmed nor disconfirmed relative to H′. Calibration may be understood as a specific aspect of this probability updating due to new evidence: the updating of the probabilities for the model-instance hypotheses associated with each base model. For instance, assume that for base model *M*, the prior probabilities, *Pr*, for model instances are equal. Now we learn new data. Then the ratio of probabilities, *Pr*_new,_ for the model instances of *M* is given by:

(2) $$\frac{Pr_{\text{new}}(M_{a,b,c})}{Pr_{\text{new}}(M_{d,e,f})}=\frac{Pr(M_{a,b,c}|E)}{Pr(M_{d,e,f}|E)}=\frac{Pr(E|M_{a,b,c})}{Pr(E|M_{d,e,f})}\cdot 1.$$

The above describes the calibration of base model *M* in light of new data *E*, assuming the prior probabilities for the model-instance hypotheses are equal.

In Steele and Werndl ([2013]), we showed why the Bayesian framework does not generally accord a special role for use-novel data, and that the no-double-counting rule does not generally hold (for a similar underlying analysis, see Howson [1988]). In short, all data should be used for calibration; in other words, the probabilities for model-instance hypotheses should be updated in light of all new data. Moreover, it is logically possible that calibration in this sense also coincides with incremental confirmation of a base model relative to another (set of) base model(s). This will occur when the likelihood ratio for the base model relative to the other (set of) base model(s) is positive. Note that the likelihood for any set of hypotheses (for example, a base model or set of base models) depends on both the likelihoods of the individual hypotheses in the set, and the prior probabilities for these individual hypotheses, conditional on the set being true. Note also that, as discussed in detail in Steele and Werndl ([2013]), the points about use-novelty and double-counting carry over to non-comparative confirmation, namely, when the concern is not the comparison between two base models but rather whether a base model is confirmed by the data *tout court*.

The intuitive position on use-novelty and double-counting clearly does not accord with the logical possibilities of Bayesian confirmation. One might nonetheless argue that the Bayesian approach does capture the spirit of the intuitive position, at least for a restricted set of cases. These special cases may indeed have been the original impetus for the intuitive position; they are cases of deterministic rather than indeterministic or stochastic hypotheses. Base models *M* and *N* both describe sets of stochastic hypotheses due to the probabilistic error term for each. As such, none of the model-instance hypotheses—that is, the instances of these base models—can ever be falsified; rather, calibration is an ongoing process whereby model-instance hypotheses are continually updated in light of new data. For a deterministic base model, by contrast, calibration may come to an end when only one instance of a base model remains unfalsified. This permits a distinction between data needed for calibration and use-novel data that may bear on the truth of a base model once calibration has finished.^2^ For cases where there is a certain symmetry in the prior probability distribution (specifically, where the likelihood of the calibrating data is the same for all base models under consideration), it may moreover be true that the data used for calibration do not change the probability of the base models, whereas the ‘use-novel’ data do (dis)confirm the base model(s).

Let us consider an example of the above special case. Assume we are comparing two base models, M′ and N′, where these models are defined as per *M* and *N* above, but without the stochastic error terms. Assume too that each of the model-instance hypotheses, whether they are associated with M′ or N′, have equal prior probability. Given that the two base models correspond to an equal number of model-instance hypotheses, the base models themselves consequently have equal prior probability. Now three data points suffices to falsify all but one hypothesis for each base model, if the data are in fact consistent with one hypothesis for each base model. Assume that three data points are learnt. For instance, the data points may be (z=6;x=1;y=2), (z=8;x=2;y=3)_,_ and (z=12;x=2;y=4). This suffices for calibrating the base models without changing their probabilities. For the data described, the unfalsified model instance for each base model is then

$$\begin{array}{l} M\prime :z(x,y)=-2x+4y+0,\\ N\prime :z(x,y)=-\frac{6}{7}\cdot x+\frac{4}{7}\cdot y^{2}+4\frac{4}{7}. \end{array}$$

Now assume that a fourth data point is learnt: (*z* = 10; *x* = 3; *y* = 4). This use-novel data point is consistent with M′ but not N′, so M′ is maximally confirmed relative to N′, which is falsified.

For the special example just described, only the use-novel data (dis)confirm the base models. So the data used for calibration are not also used for confirmation. This is due to the special structure of the prior probability function. Note also that for the special example, the number of parameters plays a key role in the sense that when there are *p* free parameters, *p* data are needed to determine the parameter values and only the rest of the data (dis)confirm the base models. (This role for the number of parameters is often emphasized by defenders of the intuitive position, for example, in (Worrall [2010]).) So we can at least say that Bayesian logic accords with the intuitive position for a restricted set of cases where the prior probability distribution has a special structure, including a deterministic relationship between hypotheses and evidence. In this way, one might argue that the intuitive position supports a Bayesian approach to model selection in that it reflects Bayesian reasoning, at least for a restricted set of cases. It follows that the intuitive position should be refined and extended in line with Bayesian logic.

Note that Schurz ([2014]) can be understood as offering a refinement of this sort (he takes a much more charitable view of Worrall’s position, interpreting use-novelty so that the role Worrall attributes to it is consistent with Bayesian reasoning). Schurz argues that the examples put forward by Worrall to motivate use-novelty and the no-double-counting rule are all of the same kind. Namely, these are cases where a base theory can be ‘successfully fitted’ to every possible set of data points, and hence fitting to some particular set of data does not raise the probability of the base theory, as the likelihood for these data is the same as the likelihood for competing possible data.^3^ In these cases, data used for calibration are not use-novel and so cannot confirm. In other cases, however, where a base theory would not be equally well fitted to any possible data, the relative success of the fit at hand may (dis)confirm the base theory.^4^ Schurz effectively refines the notion of use-novelty such that in the cases where, by Bayesian reasoning, a base theory is (dis)confirmed by its fit with the data, use-novelty is respected, and thus the ‘spread of confirmation’ does not amount to double-counting. As Schurz puts it, use-novel data bear on that part of the content of the base theory that transcends or goes beyond the data itself.

We will see in the next section that classical hypothesis-testing has similar implications to the Bayesian approach with respect to use-novelty and double-counting (at least on our interpretation of these terms) so, arguably, this method has just as much claim to clarifying and reforming the intuitive position. Later, we will consider rather different readings of the intuitive position, and what it purportedly gets right about model selection.

## 4 Classical Tests *vis-à-vis* Intuitions

Classical statistics offers various methods for assessing the reliability of model predictions. The simplest of these is, arguably, the standard hypothesis-testing method, our focus in this section. Like the Bayesian approach to model calibration and prediction, classical hypothesis testing involves the enumeration of all plausible model-instance hypotheses associated with a base model. Generally, only one base model is considered, although this includes any nested base models (as these are just subsets of the full set of model-instance hypotheses, restricted to those with common zero-valued parameters). Also in common with the Bayesian approach, calibration is not distinct from inference; calibration is just ordinary hypothesis testing, but for a specific kind of hypothesis, namely, the set of model-instance hypotheses associated with a base model.

Classical hypothesis testing differs from the Bayesian approach in that confidence in the predictions entailed by a model-instance hypothesis is not grounded in confidence or support for the hypothesis per se, but rather depends on confidence in the long-run properties of the testing procedure that is used to discriminate the hypotheses on the basis of the data (with sample-size *n*). The typical procedure is as follows: All model-instance hypotheses for which the *n* data at hand are too unlikely are rejected, leaving some subset of model-instance hypothesis that has not been rejected. These remaining accepted hypotheses (under normal conditions, a convex set) effectively form a confidence interval of plausible parameter values for the base model. The long-run properties of the testing procedure that are of interest are the type-I errors for rejecting hypotheses, and the corresponding confidence level for the set of accepted hypotheses. The type-I error is the (long-run frequentist) probability of rejecting any given model-instance hypothesis when it is in fact true; it matches the probability that is used as the cutoff for rejection (referred to as the significance level, typically 0.05 or 0.01).^5^ The confidence level is the flip-side of the type-I error/significance level; the two values add to one. The confidence level gives the (long-run frequentist) probability that the set of accepted model hypotheses—or, in other words, the confidence interval for the various parameter values—contains the true hypothesis/parameter values, if the same experiment (with *n* data generated by nature) were repeated indefinitely. The assumption here is that the set of hypotheses under consideration form a suitable continuum and the true hypothesis is indeed amongst them.

By way of illustration, assume that we need to make well-supported model predictions on the basis of model *M*, defined as in the previous section:

M:z(x,y)=ax+by+c+N(0,σ).

Assume here that *a* and *b* are unknown, but *c* and *σ* have fixed values; so there are only two free parameters. For the hypothesis-testing method, a continuous set of model-instance hypotheses is appropriate, effectively treating the unknown parameter values as unbounded reals. As before, each of the model-instance hypotheses of *M* claims to give the value of *z* that would be observed in the real world, or rather a probability distribution for *z*. Note that in the course of testing base model *M*, two simpler nested base models are also implicitly under consideration. These are:

$$\begin{array}{l} M_{x}:z(x,y)=a\cdot x+c+N(0,\sigma ),\\ M_{y}:z(x,y)=b\cdot y+c+N(0,\sigma ). \end{array}$$

Given the observed *n* data points, the hypothesis-testing method proceeds as follows: A significance level is selected, say, 0.05. The model-instance hypotheses that would not be rejected, given the data and the nominated significance level, are then identified. The structure of the hypothesis space and error term here are such that this will yield a convex set of model-instance hypotheses, or rather, convex confidence intervals for the values of the two parameters, *a* and *b*, and ultimately for values of *z*(*x*, *y*). The confidence intervals that are derived in this way are justified in the sense that, were this procedure implemented indefinitely (for different *n* data-points randomly generated by nature), the confidence interval of accepted hypotheses would contain the true hypothesis in 95% of cases (assuming that the true data-generating process is indeed described by some instance of base model *M*).

Let us reflect now on the question of use-novelty and double-counting. The analysis is similar in many respects to that in Section 3 above. Calibration just is the process of rejecting/accepting hypotheses, so it involves the confirmation of model-instance hypotheses. Moreover, in the process of calibration, one base model may be confirmed relative to an otherwise nested base model.^6^ For instance, assume that for model *M* above, hypothesis testing against the data yields 95% confidence intervals for parameters *a* and *b*, where neither of these intervals contain zero. This effectively confirms the base model *M* (with non-zero parameters *a* and *b*) relative to the otherwise nested simpler models *M_x_* and *M_y_* (for which *a* or *b*, respectively, equals zero). Thus, although the logic of confirmation is different, the importance of use-novel data in classical hypothesis testing is as per the Bayesian method, namely, it is not a relevant consideration. Moreover, as just illustrated, the same data that are used for calibration may also confirm one base model relative to another one, contrary to the no-double-counting rule.

Contrary to our comments here, one might argue that Worrall’s maxims are either upheld or else they are inapplicable, in the case of comparing only nested base models, as per classical hypothesis testing. There is, of course, a sense in which the fully inclusive base model, here *M*, can neither be confirmed nor disconfirmed by any data used for calibration, because *M* is assumed to be true. So calibration does not involve a ‘spread’ of confirmation to *M*; only specific instances of *M* (or, as we put it, sets of these instances of *M*—that is, the nested base models) can be (dis)confirmed relative to other (sets of) specific instances of *M*. This is, however, hardly a strong vindication of Worrall’s use-novelty and double-counting maxims: the maxims are trivially upheld by classical hypothesis testing if we are interested in the (dis)confirmation of the fully inclusive base model, because the method does not have the resources for assessing this particular base model. Schurz ([2014]) would presumably take a different tack, arguing that the question of use-novelty is inapplicable in the case of hypothesis testing because it is not the case that the competing base models can all be successfully fitted to any possible data set. Indeed, in our example above, the simpler base models do not successfully fit the data. Note, however, that a lot hangs on what is understood by a ‘successful fit’ here; it is, in fact, a consequence and not an assumption of hypothesis testing that the simpler base models do not ‘successfully fit’ the data in our example and so are disconfirmed by these data relative to *M* with non-zero parameters *a* and *b*. (After all, the data are not logically inconsistent with the simpler base models.) Our own view is that the lack of successful fit for these simpler base models is another way of saying that these base models are disconfirmed in the process of calibration, contrary to the intuitive position.

Finally, we note that Mayo (see, for instance, [1991], [2014]) also casts doubt on the significance of use-novelty for classical or ‘error’ statistics. Mayo argues that what matters in determining whether good fit with data has confirmatory power is not use-novelty or double-counting *per se*, but rather whether the procedure for fitting the base theory to evidence (‘the use-constructed procedure’) violates the severity requirement. She claims that severity is often violated in the case of use-construction, but it is not always violated, as in the construction of confidence intervals described above. Arguably, Mayo is talking past Worrall to some extent, in that she is not concerned with incremental confirmation of one base theory relative to another competing base theory; furthermore, it is not clear whether her severity requirement succeeds in discriminating the cases that she intends. (This is queried in (Hitchcock and Sobe [2004]), with responses from Mayo in her ([2008], [2014]) articles). In any case, we agree with Mayo that use-novelty does not play a primary role in classical statistics and that there are good procedures by classical standards that involve double-counting.

## 5 Classical Model-Selection Methods *vis-à-vis* Intuitions

### 5.1 Introducing classical model-selection methods

We now turn to classical model-selection methods, in particular to cross-validation and the Akaike information criterion selected for their differing approaches to use-novelty and its relation to double-counting. Let us first consider what the classical model-selection methods have in common and how they are distinct from the methods described in Sections 3 and 4 above.

All model-selection methods (interpreted broadly to include both Bayesian and classical methods) are in the business of assessing the reliability of model predictions, not least whether the predictions of one model are better supported by the data at hand than those of another model. We have already noted the difference between the Bayesian method and classical hypothesis testing: while both focus on the model-instance hypotheses associated with base models, the former is concerned with assessing the truth of these hypotheses in light of the data, while the latter is concerned with whether the procedure for selecting some hypotheses over others on the basis of the data is reliable in the long run. The classical model-selection methods introduced in this section are also concerned with procedures for making model predictions, and the long-run properties of these procedures. This is the hallmark of the classical approach. Unlike hypothesis testing, however, the procedures canvased here do not assume the truth of any particular base model (where nested base models are also of interest); rather, this is the problem to be addressed. The whole point of these model-selection methods is to provide a comparative assessment of different base-model procedures for deriving model predictions on the basis of data.^7^

The base-model procedures themselves are, in a sense, simpler than for hypothesis-testing. Rather than identifying a confidence interval of ‘accepted’ model-instance hypotheses (premised on the base-model being true), the procedure is instead to identify the single model-instance hypothesis that has ‘best fit’ with the data (makes the data most probable); this is referred to as the maximum-likelihood instance of the base model. Note that, as per hypothesis-testing, the sample size, or the amount of data used for selecting the maximum-likelihood model (*n*), is also part of the specification of the procedure. The base-model procedures in effect amount to calibrating the base model such that it can be used for making predictions.

The reliability of a particular ‘base-model procedure’, thus described, is taken to be its long-run average predictive accuracy, or the average distance from the truth, if repeated infinitely many times, of a prediction derived from the procedure given *n* random data points generated by nature. The problem, of course, is that the true data-generating process of nature is unknown, so the scientist cannot simply calculate which base-model procedure has the best long-run average predictive accuracy. The different classical model-selection methods effectively offer different ways of estimating this long-run average predictive accuracy of the respective base-model procedures. We will examine two of these methods. They each assign scores to base-model procedures, such that they can be ranked in terms of estimated long-run average predictive accuracy.

Before we do so, let us mention that Hitchcock and Sober ([2004]) also discuss model-selection theory. They consider various ways scientists might assess/confirm models, some involving novel or predicted data and others just accommodated data. Although some model assessments relying on accommodated data are inferior to assessments involving predicted data, the use of accommodated data may even be advantageous if the danger of over-fitting models to data is otherwise accounted for (as per some model selection methods). The focus of this article is somewhat different, in that we consider a range of prominent logics of confirmation, focusing rather on the roles that use-novelty and double-counting play. We do not presume, and indeed we do not think it obvious, that one or more of these logics of confirmation is superior to others. Moreover, note that there is a philosophical debate regarding the scope and completeness of estimators of predictive accuracy proposed in model-selection theory, such as those we discuss below (for instance, Forster [2007]; Myrvold and Harper [2002]). The issue of scope is that model-selection theory is defensible only to the extent that nature (at least, that part of nature of interest) is effectively a stationary data generation mechanism, such that all data, existing and prospective, are independent and identically distributed. The issue of completeness concerns the extent to which any single estimator of predictive accuracy assesses what some think really matters, that is, the extent the data agree on the value of causally relevant parameters. For instance, Forster ([2007]) argues that the methods from classical model selection theory are incomplete indicators of empirical success and predictive accuracy; instead, empirical success and predictive accuracy amount to a hierarchical structure that emerges from the agreement of independent measurements of theoretically postulated quantities. Here we set these further debates aside. We simply explore what role use-novelty and double-counting play if one takes classical model-selection theory as providing a good measure of predictive success and confirmation (many think that, at least in certain situations, this is the case).

### 5.2 Two cases

Case I: First, consider the method of cross-validation. The standard calibrating procedure as described above is followed for all base models under consideration. That is, for each base model, the best-fitting model instance given all *n* data is identified, and the parameter(s) corresponding to this model instance is (are) then presented as the best estimate(s) of the free parameter(s) for the base model in question.

The cross-validation estimator for the reliability of each base-model procedure (that is, each calibrated base model) is as follows: Given *n* data points, one starts by using the first *n* – 1 data points to arrive at the best-fitting model instance and then uses the remaining data point to test the performance of the model instance (by calculating the distance between the predicted data point and the actual data point). This is repeated for all possible selections of *n* – 1 data points to calculate the average distance between the predicted data points and the actual data points (with some distance measure such as the squared distance or the Kullback–Leibler discrepancy). This average distance is an asymptotically unbiased estimator of the predictive accuracy of the base-model procedure given *n* data points (Linhard and Zucchini [1984]; Zucchini [2000]). Cross-validation is a universal method because it only assumes that data are independently and identically distributed (cf. Arlot and Celisse [2010]).

Here is a simple example. Consider the Euclidean distance as a distance measure and the base models already discussed in the previous subsection:

$$\begin{array}{l} M:z(x,y)=ax+by+c+N(0,\sigma ),\\ M_{x}:z(x,y)=ax+c+N(0,\sigma ). \end{array}$$

Suppose the six data points of (*x, y, z(x, y)*) are (0, 0, 10), (100, 0, 199), (0, 100, 10), (100, 100, 210), (200, 100, 410), (100, 200, 199). For base model *M*, one starts by using the first five data points to arrive at the best-fitting model instance given these five data points, which is 1.96*x* + 0.12*y* + 2. Then one uses the remaining data point (100, 200, 199) to test the performance of the model instance by determining the distance between the predicted value, 222, and the actual value, 199, that is, 23. This is repeated for all six selections of *n* − 1 data points. The six differences are then averaged giving (32+26.6667+8.8889+10.4348+27.8261+8)/6=18.9694. Thus 18.9694 is the cross-validation estimator of the predictive accuracy of *M* given six data points.^8^ Then one turns to base model *M_x_*. The average of the six differences for model *M_x_* is: (8+15.7143+8+8.5714+26.6667+15.7143)/6=13.7778. Thus 13.7778 is the estimator of the predictive accuracy of *M_x_* given six data points. Since 13.7778 is smaller than 18.9694, the maximum-likelihood procedure for base model *M_x_* is confirmed relative to that for *M*. The maximum-likelihood instance of *M_x_* is identified, or, in other words, the best-fitting values of the parameters for *M_x_* given all six data are identified, which are *a* = 1.97647 and *c* = 5.29412. Note that this example is especially interesting because it shows that while, clearly, *M* has a better fit to the data than *M_x_* (because *M_x_* is a nested model of *M*), *M_x_* nevertheless scores better in terms of expected predicted accuracy. Note that, as already mentioned above, it is just an accidental feature of the base models *M_x_* and *M_y_* that they are nested base models. The methods of model selection work for any arbitrary comparison between base models, whether they are nested or not.^9^

For a method such as cross-validation, it is crucial that when estimating predictive accuracy the data used to test the predictions of model instances are novel. This is also emphasized by Hitchcock and Sober ([2004]) and expressed by the following quotes on cross-validation (which are made in the context of discussing statistical bias):

The results of these experiments using cross-validation reinforce the point that knowledge gained from the model building dataset is biased. (Michaelsen [1987], p. 1598)

An example of controlled division is provided by the cautious statistician who sets aside a randomly selected part of his sample without looking at it and then plays without inhibition on what is left, confident in the knowledge that the set-aside data will deliver an unbiased judgment on the efficacy of his analysis. (Stone [1974], p. 111)

Hence use-novelty is important here, just as proponents of the intuitive position would argue. However, recall that for the base-model procedure, all the data are used for calibration to find the best model instance. Hence cross-validation involves double-counting. So we see that what we find for methods such as cross-validation is different and more nuanced than the intuitive position: use-novelty is important but at the same time there is double-counting. Hence, contrary to the intuitive position, use-novelty does not imply the ‘no-double-counting rule’, which says that data used in calibrating a model cannot also play a role in confirming the predictions of the model. Despite this divergence, one might contend that cross-validation captures the spirit of the intuitive position, in that it gives significance to use-novelty when it comes to the confirmation of base models and, furthermore, the calibration step itself, where all the data are used to determine the best model instance, has no bearing on the confirmation of the base models. This could be said to accord with Worrall’s remarks on conditional versus unconditional confirmation, the latter of which requires use-novelty. Thus one might argue that the intuitive position should be refined in keeping with cross-validation.

Notice that what we have called cross-validation is often also called (*n* − 1)-cross-validation to emphasize that one data point is used to test the predictive accuracy of the procedure. There is also (*n* − *k*)-cross-validation, where *k* data points are used to test the predictive accuracy of the procedure (and, again, as a final step all *n* data are used for calibration). It is important to realize that these methods lead to biased estimates because one tests the performance of the base-model procedure when *n* – *k* data points are used for calibration and not what one would really like to test, namely, the performance of the procedure when *n* data points are used for calibration (as is actually done). There is least bias for (*n* − 1)-cross-validation, and, as mentioned above, this method yields an asymptotically unbiased estimate (Zucchini [2000]; Arlot and Celisse [2010]). Some have suggested using only half of the data for calibration and the rest for confirmation, or using only *p* data for calibration (where *p* is the number of free parameters) and the rest for confirmation (cf. Oreskes *et al.*[1994]; Worrall [2010]). Such methods indeed yield unbiased estimators of the predictive accuracy of base-model procedures that use *n*/2 or *p* data points for calibration (Linhard and Zucchini [1984]; Zucchini [2000]). However, this is not in line with model-selection theory, where the maxim is to use all the data to estimate the most plausible value of the free parameters and hence to evaluate the predictive accuracy of the base-model procedure when all data are used for calibration (because one does not want to throw away any information about the parameters).

Case II: The Akaike information criterion for finite sample sizes measures the distance between the simulated and actual observations in terms of the Kullback–Leibler discrepancy.^10^ Here again the base-model procedures under consideration are the standard variety: the best-fitting model instance for each base model relative to the *n* data points is identified. This is the calibrated base model that would be used for prediction.

By way of estimating the long-run average predictive accuracy of the aforementioned procedure for each base model, one first calculates the discrepancy between the maximum-likelihood model instance and the actual data points. This discrepancy amounts to $-\frac{\text{ln}[L]}{n}$, where *L* is the maximum value of the likelihood function (Zucchini [2000], pp. 52–3). This is then used to calculate the confirmation score of the procedure given *n* data points as follows:

(3) $$C_{AICc}=-\frac{\text{ln}[L]}{n}+(\frac{p}{n}+\frac{p(p+1)}{n(n-p-1)}),$$

where *p* is the number of free parameters. It can be shown that *C_AICc_* is an unbiased estimator of the discrepancy between the true data generation mechanism and the base-model procedure, that is, the average predictive accuracy of the base-model procedure (Burnham and Anderson [1998]; Linhard and Zucchini [1984]). For *C_AICc_*, the data also have to be independently and identically distributed (and there are some further technical assumptions; see Burnham and Anderson [1998]; Linhard and Zucchini [1984]).

Let us illustrate the Akaike information criterion with the simple base-models *M* and *M_x_* above. It can be shown that for normally distributed error terms with a constant variance, *C_AICc_* can be easily computed:

(4) $$C_{AICc}=\frac{\text{ln}[2\pi {\hat{\sigma }}^{2}]+1}{2}+(\frac{p}{n}+\frac{p(p+1)}{n(n-p-1)})$$

where ${\hat{\sigma }}^{2}=\sum_{i=1}^{n}{\hat{ɛ}}_{i}^{2}/n$, with ${\hat{ɛ}}_{i}$ being the estimated residuals in a linear regression with the Euclidean distance measure (Burnham and Anderson [1998], p. 63). Starting with *M*, one first determines the best model instance given all data points, which is 1.98182*x* − 0.0181818*y* + 6.36364. Then one calculates the sum of the squared residuals and divides it by six, which amounts to $(3.6364^{2}+14.5455^{2}+5.4546^{2}+7.2727^{2}+9.0909^{2}+10.9091^{2})/6=84.8488$. By plugging this into Equation (4), one obtains (*p* = 3 because the estimated parameters are *a*, *b*, *c*): (ln[2π84.8488]+1)/2+3/6+(3*4)/6(6−3−1)=5.13937, which is the expected predictive accuracy for the maximum-likelihood instance of *M* given six data points. Then the same is repeated for *M_x_*. That is, one determines the best model instance given all data points, which is 1.987647*x* +5.29412, and calculates the sum of the squared residuals and divides it by six, which is $(4.7059^{2}+12.9412^{2}+4.7059^{2}+7.0588^{2}+9.4118^{2}+12.9412^{2})/6=86.2748$. From Equation (4), one obtains (*p* = 2 because the parameters that are estimated are *a* and *c*): (ln[2π86.2748]+1)/2+2/6+(2*3)/(6(6−2−1))=4.31437, which is the expected predictive accuracy for the maximum-likelihood instance of *M_x_* given six data points. Since 4.31437 is smaller than 5.13937, *M_x_* is confirmed over *M*, and the best estimates for the parameter values of *M_x_* are *a* = 1.987647 and *c* = 5.29412. Note that, again, *M_x_* is confirmed over *M*, even though *M* has a better fit with the data than *M_x_* (because *M_x_* is a nested model of *M*).

Clearly, for methods such as AICc, there is double-counting because all the data are used to estimate the value of the free parameters (the base-model calibrating procedure) and also to calculate the confirmation score (Equation (3)). Further, the data used for confirmation are not use-novel because the maximum likelihood given all the data is a key term in the confirmation expression; unlike cross-validation, there is no apparent assessment of how the base-model procedure fares on new data. Still, in a precise sense there is a penalty term in the expression for the degree of confirmation because the data have already been used for calibration.

To show this, let us compare two methods for evaluating the predictive accuracy of base-model procedures that use *n* data for calibration and only differ because (i) in the first case, the data used for confirmation are use-novel, and (ii) in the second case, they are not (where we work with the Kullback–Leibler divergence as distance measure). In case of (i), one starts by using *n* data for calibration to find the model instance that fits the data best. Then one goes on to use other novel *n* data points to calculate the distance between the predicted and actual data points to estimate the predictive accuracy of the procedure (the reason for choosing *n* data points is that we later want to compare this method with the Akaike information criterion for finite sample sizes, which also uses *n* data points for confirmation).^11^ One can show that this estimator is unbiased (Linhard and Zucchini [1984]; Zucchini [2000]).

Let us now turn to (ii) and AICc. One starts as in (i) and uses *n* data points for calibration to find the best model instance. But now one does not test the performance of the model instance with novel data points but instead with the same *n* data points that have been used for calibration. With these data points, one does exactly what has been done in (i) and calculates the average Kullback–Leibler divergence between the best-fitting model instance and the *n* data points. What one obtains is the term on the left-hand side of *C_AICc_* (Equation (3)). What has been done so far is exactly as in (i), with the only difference being that the data used are not use-novel. But the term on the left-hand side of *C_AICc_* is not enough—using just this term would lead to an estimate that is statistically very biased. Intuitively speaking, the estimate based on maximum-likelihood fit is a bit optimistic because the good fit of the best-fitting model is specific to the data at hand, rather than to a new piece of data and hence the procedure is statistically biased (tending to overestimate predictive accuracy in the long run). So in order to get an unbiased estimator of the predictive accuracy of the procedure when *n* data are used for calibration, we also need the term on the right-hand side of *C_AICc_*. Hence this term on the right-hand side can be interpreted as a penalty term because the data have already been used before for calibration. Because of this penalty term, use-novelty still plays a certain role (but one that is more nuanced than the role it plays in the standard intuitive account).^12^ Thus the AIC method could also be argued to be an apt refinement of the intuitive position in that use-novelty matters for comparing base models.

Finally, recall that defenders of the intuitive position often emphasize the crucial role of the number of parameters, *p*. For the second case (ii), the number of parameters also plays a role, but it differs from the one advocated by the intuitive position. According to the intuitive position, the number of parameters, *p*, is crucial because it tells one that *p* data points should be used for calibration and the rest for confirmation. In contrast to this, in model-selection theory, all data should always be used for calibration. Still, for case (ii) and methods such as AICc, the number of parameters plays a role in the sense that the penalty term, $\left(\frac{p}{n}+\frac{p(p+1)}{n(n-p-1)}\right)$, depends on the number of parameters of the procedure.

There is an important connection between cross-validation and the Akaike information criterion. Namely, it can be shown that the (*n* − 1)-cross-validation estimator is asymptotically equivalent to the Akaike information criterion— that is, their estimates of the predictive ability of models coincide approximately as *n* goes to infinity (Stone [1977]). Therefore, in some sense, use-novelty is also implicitly contained in the Akaike information criterion. Our discussion can make sense of this in the following way: while for cross-validation, use-novelty is an important requirement, for the Akaike information criterion, use-novelty also plays some role in the sense that there is a penalty term for the Akaike information criterion because the data have already been used for calibration.

## 6 Re-examining Our Case Study

Let us now re-examine our case study in light of the discussion of the previous sections and ask whether Willett *et al.* proceed according to any of the four approaches discussed in this article. A closer look reveals that they follow the framework of classical tests as outlined in Section 4. Recall that for base model *M*_1_, there are two free parameters—one that measures the extent of the response to the anthropocentric forcings and one that measures the extent of the response to the natural forcings. For base model *M*_2_, there is only one free parameter—this measures the extent of the response to the natural forcings (the unknown parameter values are treated as taking on some value in the reals). Willett *et al.* first consider base-model *M*_1_ and use the data about surface specific humidity to identify the model-instance hypotheses that would not be rejected, given the significance level of 0.05. In this way, they arrive at confidence intervals for the anthropogenic forcing and natural forcing, respectively. Given that neither interval contains the value zero, this effectively confirms base model *M*_1_ (with positive values for both free parameters) relative to the nested simpler base model, *M*_2_ (for which the anthropocentric forcing would be zero). As is the case for classical tests, for Willett *et al.*, use-novelty is not a relevant consideration and the same data are used both for calibration and for confirmation of base model *M*_1_ relative to *M*_2_.^13^

While Willett *et al.* appeal to a classical and not a Bayesian framework, their procedure nevertheless could be roughly reconstructed in Bayesian terms as follows: Let the uncertainty about the forcing values be represented by some prior probability distribution over the possible forcing values conditional on base-model hypothesis *M*_1_—say, a uniform distribution over the possible forcing values. This prior will also incorporate instances of nested base model *M*_2_. For a uniform prior, the posterior probability distribution over model-instance hypotheses (the result of calibration) will mirror the relative likelihoods of these model instances. Here, the relative likelihoods depend on fit with data, *E*, about changes in surface specific humidity. Willett *et al.* find that the best-fitting forcing values for *M*_1_ are (1.12, 2.22). In effect, this model-instance hypothesis has highest posterior probability under a uniform prior. Moreover, given the classical 95% confidence intervals, we know that instances of nested base model *M*_2_ are not amongst the model instances that have best fit with the data nor the highest posterior probability on the assumption of a uniform distribution. Indeed, it is plausible that the likelihood ratio for base-model hypothesis *M*_1_ relative to nested hypothesis *M*_2_ is greater than one. So we see that the Bayesian reconstruction of Willett *et al.*’s logic yields the same conclusion *vis-à-vis* the intuitive position, namely, in contrast to this position, all the data are used for both confirmation and calibration, and the data used for confirmation are not use-novel.

Willett *et al.* subscribe to the classical framework of hypothesis testing outlined above, which is different from the framework of classical model-selection theory. Both frameworks demand double-counting, since all data must be used for calibration. But of course use-novelty only plays a role in the latter framework. Willett *et al.* do not use any method, such as cross-validation, where use-novelty plays a role, nor a method such as AICc, where there is some penalty term because the data have already been used for calibration or, more generally, where it is somehow taken into consideration that it is easier for model *M*_1_ to provide a good fit with the data than for nested model *M*_2_. Instead, Willett *et al.* simply use all the data for calibration and confirmation, and use-novelty plays no role for them. Hence their view on double-counting and use-novelty is not the more nuanced view presented by classical model-selection theory.

Let us briefly explain how the analysis would go if a model-selection method such as cross-validation were used. First, one would consider model *M*_1_ and use the first *n* – 1 data points to determine the best-fitting model instance of *M*_1_, and then use the remaining data point to calculate the distance between the predicted and actual data point. This would need to be repeated for all possible selections of *n* – 1 data points to calculate the average distance between the predicted and the actual data points for model *M*_1_ (with some distance measure such as the squared distance or the Kullback–Leibler discrepancy). Exactly the same would need to be repeated for model *M*_2_ to calculate the average distance between the predicted and actual data points for model *M*_2_. Then one would compare the average distance between predicted and actual data points for *M*_1_ and *M*_2_, and the model procedure with the lower score would be confirmed relative to the model procedure with the higher score. It is not entirely clear what the model procedure with the lower score would be; one would need to do the calculations to know for sure. Yet it seems likely that the model with the lower score will still be *M*_1_ because the amount of data is very large, *M*_1_ has only one additional parameter when compared to *M*_2_, and all model instances of *M*_2_ have a bad fit with the data (while there are model instances of *M*_1_ that fit to the data well). Assuming that *M*_1_ is indeed confirmed over *M*_2_, as a final step all the data points would be used for calibration to obtain the parameter values that correspond to the best-fitting model instance of *M*_1_; the values obtained in this way would be (1.12, 2.22) (as in Willett *et al.*).

To conclude, Willett *et al.* adopt the classical framework of hypothesis testing, where use-novelty is not a relevant consideration and double-counting is legitimate. Hence their view is not in line with the intuitive position, which argues for use-novelty and against double-counting; neither is it with the more nuanced account presented by model-selection theory. While Willett *et al.* do not adopt a Bayesian framework either, their views on use-novelty and double-counting coincide with the Bayesian position, where double-counting is proper and use-novelty is not a relevant consideration.

In general, when climate scientists worry about the legitimacy of double-counting and whether data should be use-novel, an important insight from our discussion is that an answer to these worries depends on the confirmatory framework. In this article, we have considered three major confirmation frameworks: In the Bayesian framework and the classical testing framework, double-counting is proper and use-novelty is beside the point. In classical model-selection theory, double-counting is also proper, but the role of use-novelty is more nuanced. Either data are required to be use-novel or there is a penalty term because the data have already been used for calibration. Hence if climate scientists worry about double-counting and use-novelty, they should carefully think about which confirmation framework they would like to adopt. This in turn will lead them to certain conclusions about double-counting and use-novelty.

## 7 Conclusion

Where does all this leave the general dilemma with which we started? We noted at the outset that in prominent examples from the history of science, use-novel as opposed to accommodated data intuitively provide more compelling support for the reliability of a theory. The puzzle is how to explain these intuitions about use-novelty and confirmation such that other deeply held intuitions or principles concerning confirmation are also respected. To this end, Worrall makes an important conceptual move: He equates accommodating data to settling on a refined version of a general theory, or, in more technical language, to calibrating the free parameters of a base theory or model. We have seen, however, that the intuitive position Worrall endorses regarding use-novelty and double-counting with respect to the calibration of free parameters is not in fact a widespread truth that is common to all the major logics of confirmation.

Indeed, none of the prominent logics of confirmation endorses the intuitive position in full, that is, that use-novel data are not just special for confirmation—in fact, only data not used for calibration can confirm a general theory. The troubles arise for stochastic theories or models, which are, moreover, widespread in scientific practice, as illustrated by our example from climate science. The question then arises as to which confirmatory framework best underpins and advances the intuitive position. We showed that the Bayesian or classical hypothesis-testing framework does capture a certain simplicity in the intuitive position that holds for special deterministic cases: use-novel data are identified as data not used for calibration, and only these data can (dis)confirm a base model or theory. The so-called classical model-selection methods instead respect the intuitive position in a different way: use-novelty has a special role to play in confirmation, or in assessing the reliability of model predictions, but use-novel data cannot simply be identified as data not used in calibration. We are thus left with a quandary. How it is resolved will depend on one’s broader commitments regarding the distinguishing properties of the various logics of confirmation.

## Footnotes

^1^ Note that the main claims are already articulated in (Worrall [1985], [1989]; see also his [2002], [2006]). The position is further clarified in (Worrall [2014]).

^2^ Note that here we are taking Worrall quite literally in his characterization of ‘use-novelty' and ‘double-counting'. For instance, Worrall ([2014], p. 55) states that ‘what matters is whether or not the evidence was used in the construction of the theory (or rather the particular version of the theoretical framework/program that entails it)'.

^3^ We take it that Schurz means by ‘successfully fitted' that the distribution of likelihoods for the various instances of the base theory, as well as the corresponding distribution of prior plausibility for the instances, conditional on the truth of the base theory, are the same for all possible data sets. Only in this case does it follow that the likelihoods of all possible data with respect to some base theory are equal.

^4^ Strictly speaking, whether there is (dis)confirmation of the base theory depends on how the likelihood for the theory compares with that of other base theories.

^5^ To be more precise, a model-instance hypothesis is rejected if the data lie in the rejection region for the hypothesis, where this region includes the most extreme (roughly, furthest away) data relative to the hypothesis that together have a likelihood equivalent to the significance level.

^6^ Here it is really the case that the base model subtract the nested model is (dis)confirmed relative to the nested model.

^7^ Elsner and Schmertmann ([1994]) similarly emphasize that classical model-selection methods assess procedures for identifying a model instance for prediction rather than the model instances themselves. Using their terminology, the targets for confirmation are ‘algorithms' rather than ‘base-model procedures'.

^8^ To be precise, 17.969 is the estimate of the predictive accuracy of the *M*-procedure, where this procedure amounts to finding the maximum-likelihood instance of *M* based on six data points.

^9^ For instance, one could equally compare the performance of base models *M* and *N* of Section 3 (which are not nested).

^10^ Our conceptual points also hold for other distance measures.

^11^ The method would also yield an unbiased estimate of predictive accuracy of the procedure if one used any other finite number of novel data points. The more data points, the better, in the sense that the more data points are used, the smaller will be the variance of the estimator.

^12^ Note that Hitchcock and Sober ([2004]) also point out that for *CAICc* use-novelty (or rather double-counting) is not a relevant consideration, but our claim about the penalty term is novel and has not been made by them.

^13^ Again, note that really *M*_1_ subtract *M*_2_ is confirmed relative to *M*_2_.
